# A multi-stage SEIR(D) model of the COVID-19 epidemic in Korea

**DOI:** 10.1080/07853890.2021.1949490

**Published:** 2021-07-16

**Authors:** Hee-Young Shin

**Affiliations:** Department of Economics, Raj Soin College of Business, Wright State University, Dayton, OH, USA

**Keywords:** COVID-19, epidemic model, the SEIR(D) model, non-pharmaceutical interventions (NPIs), (South) Korea

## Abstract

**Background:**

This paper uses a SEIR(D) model to analyse the time-varying transmission dynamics of the COVID-19 epidemic in Korea throughout its multiple stages of development. This multi-stage estimation of the model parameters offers a better model fit compared to the whole period analysis and shows how the COVID-19’s infection patterns change over time, primarily depending on the effectiveness of the public health authority’s non-pharmaceutical interventions (NPIs).

**Methods:**

This paper uses the SEIR(D) compartment model to simulate and estimate the parameters for three distinctive stages of the COVID-19 epidemic in Korea, using a manually compiled COVID-19 epidemic dataset for the period between 18 February 2020 and 08 February 2021. The paper identifies three major stages of the COVID-19 epidemic, conducts multi-stage estimations of the SEIR(D) model parameters, and carefully infers context-dependent meaning of the estimation results to help better understand the unique patterns of the transmission of the novel coronavirus (SARS-CoV-2) in each stage.

**Results:**

The original SIR compartment model may produce a poor and even misleading estimation result if it is used to cover the entire period of the epidemic. However, if we use the model carefully in distinctive stages of the COVID-19 epidemic, we can find useful insights into the nature of the transmission of the novel coronavirus and the relative effectiveness of the government’s non-pharmaceutical interventions over time.

**Key messages**
- Identifies three distinctive waves of the COVID-19 epidemic in Korea.
- Conducts multi-stage estimations of the COVID-19 transmission dynamics using SEIR(D) epidemic models.
- The transmission dynamics of the COVID-19 vary over time, primarily depending on the relative effectiveness of the government’s non-pharmaceutical interventions (NPIs).
- The SEIR(D) epidemic model is useful and informative, but only when it is used carefully to account for the presence of multiple waves and context-dependent infection patterns in each wave.

## 1. Introduction

The SIR model of epidemic, Kermack and McKendrick’s seminal compartment model [1] has been widely used to analyse various epidemics, and the ongoing COVID-19 pandemic is not an exception (See, for example, [2–5]). This paper explores how well one of its variants, the SEIR(D) model, fares with the current COVID-19 epidemic data for South Korea (‘Korea’ hereafter).

The original SIR model assumes that (1) the susceptible population is relatively homogeneous, and that (2) parameters used in the model remain invariant throughout the entire epidemic period. In the real-world, however, the susceptible population is not homogeneous. Nor is it that the one-time transmission dynamics captured by the estimated model parameters stay constant throughout the whole period of observation. More importantly, the public health authority’s non-pharmaceutical interventions (NPIs), even in the absence of vaccines and medical treatments, can significantly alter the value of parameters, drastically changing the transmission dynamics of the COVID-19 epidemic. [6,7]

Given this real-world complexity, to what extent one can safely rely on existing SIR-based epidemic models to analyse the COVID-19 pandemic becomes a controversial issue. This paper addresses this problem by demonstrating that carefully calibrated epidemic models used in a particular epidemiological context can better capture potentially time-varying and context dependent parameters. These context dependent and time-varying parameters can then be used to evaluate the relative effectiveness of non-pharmaceutical policy interventions to the COVID-19 in each stage. The analysis in this paper can offer a useful insight into both developing a better theoretical model and evaluating existing public health policies.

To demonstrate this point, the paper begins with a brief overview of the COVID-19 epidemic in Korea. This sub-section serves as a useful reference for interpreting later empirical estimations of the SIR-based model parameters. The next sub-section introduces the SEIR and SEIRD epidemic model, data, and methods that we employ in this paper. The third and fourth section reports the result of the statistical analysis, together with a careful interpretation of the estimation results, in order. The last section concludes the discussion by drawing some implications.

## 2. The context, models, methods, and data

### 2.1. An overview of the COVID-19 epidemic in Korea

Since the first imported case was detected in late January of 2020[8], there have been three distinctive phases of the COVID-19 epidemic in Korea. The first full-blown spread of the novel coronavirus began in late February. According to KCDC, this first wave is triggered by a massive religious assembly of a particular Christian cult, known as Shincheonji Church of Jesus. [9] The novel coronavirus quickly spread among those who attended this religious gathering, which was held in a tightly packed mega church and other religious buildings. This first wave lasted until early-May (May 10), when the new daily confirmed case fell below the weekly average of 50.

The second wave of the COVID-19 epidemic began in early August, as the number of confirmed cases rose sharply from weekly average of less than 50 to a peak of 441 on 28 August 2020. The immediate trigger of this second spike was also related with another super spreader event that was more political in nature: A conservative opposition party and some Christian fundamentalist factions joined their forces to hold a massive political demonstration at the centre of the capital city, Seoul, blaming the government’s various epidemic mitigation strategies. Unlike the first wave, however, the public health authority was unable to implement proper public healthcare measures such as enlisting and conducting pre-emptive diagnostic testing for suspected patients who participated in the political rally. Leading figures of Christian fundamentalist movements fiercely opposed the public heath authority’s healthcare measures and even instructed their members not to fully cooperate with the authority. Consequently, it took much longer time for the Korean health authority to bring down the number of daily confirmed cases below 100 (only by 20 September 2020), and it is not even clear whether the second wave was suppressed at all.

The third and concurrent wave of the COVID-19 epidemic began around mid-October with daily confirmed cases rising from the low 60 s to the peak of 1241 on 25 December 2020. Compared to the previous two waves, the latest phase of the infection dynamics does not seem to be associated with any single super spreader event. Instead, it stems from persistent small scale and multi-sited infection cases found in childcare and elderly care facilities as well as private education, various entertainment facilities and religious venues throughout the country. The median age of newly confirmed cases is also lower than during the second wave, as increasing numbers of younger asymptomatic patients are suspected to spread virus variants that are likely to be more infectious and deadlier to some demographic groups.

During the whole tumultuous period of this epidemic, the Korean public health authority led by the Central Disease Control and Management Headquarter has maintained and implemented consistent non-pharmaceutical interventions and proactive public health-care measures. The health authority has adopted policies of (1) conducting pre-emptive and targeted PCR-based diagnostic testing on a massive scale, (2) tracing epidemiological links of confirmed patients, fully utilising the information-communication technology infrastructure, and (3) expanding public and private medical facilities and equipment to accommodate the need of quarantining and treating different groups of patients in accordance with the severity of clinical symptoms (See also [9]).

**Figure 1.**
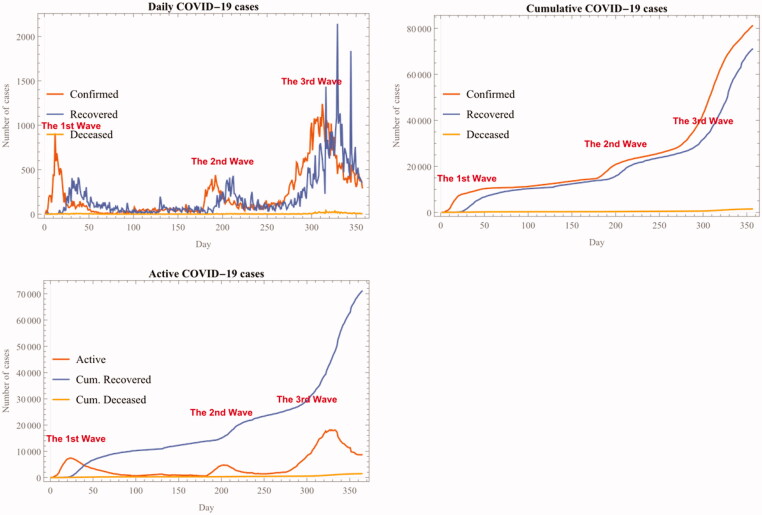
Daily, cumulative, and active COVID-19 cases in Korea, 18 February 2020–08 February 2021.

The following figure shows these three distinct phases of the COVID-19 epidemic from 18 February 2020 (Day 1) to 08 February 2021 (Day 360). The ‘Active’ case in the third chart in the figure represents the number of confirmed cases minus the sum of both recovered and deceased cases (See Figure 1):

### 2.2. The proposed SEIR(D) models

The goal of this paper is to analyse the unique pattern of the novel coronavirus infection in each phase of the COVID-19 epidemic using both SEIR and SEIRD model. These two models are a slight variation of Kermack and McKendrick’s original SIR(D) model. The SIR compartment model classifies the homogeneous population into its sub-groups, namely, the susceptible, the infected, and the recovered population and traces how each population group interacts with one another over time. Ignoring so-called ‘vital’ dynamic variables such as the natural birth and death rate, we can write down the SIR model as a system of three differential equations of the form:

(1) $$\begin{array}{c} s^{\prime }\left[t\right]=-\alpha i\left[t\right]s\left[t\right]\\ i\prime \left[t\right]=\text{ αi}\left[t\right]s\left[t\right]-\text{ βi}\left[t\right]\\ r\prime \left[t\right]=\text{ βi}\left[t\right] \end{array}$$

where s(t), i(t), and r(t), represents the number of susceptible, infected, and recovered population at time t, respectively. The parameters, α and β then represents the transmission rate (or infection rate) and the recovery rate. These two parameters jointly determine the rate of change in the number of infected and recovered population among the susceptible population.

If we include the number of deaths associated with the virus infection into this model, the outcome is the SIRD model, where D represents another compartment of the population, the deceased group with the corresponding parameter γ > 0 that represents the death rate (fatality rate) associated with the virus infection. The SIRD model has the following four differential equations with three unknown parameters:

(2) $$\begin{array}{c} s\prime \left[t\right]=-\alpha i\left[t\right]s\left[t\right]\\ i\prime \left[t\right]=\text{ αi}\left[t\right]s\left[t\right]-\text{ βi}\left[t\right]-\text{ γi}\left[t\right]\\ r\prime \left[t\right]=\text{ βi}\left[t\right]\\ d\prime \left[t\right]=\text{ γi}\left[t\right] \end{array}$$

Building upon both SIR and SIRD model, an epidemiologist can further develop a slightly more complex model such as the SEIR(D) model in order to account for the prior exposure to the virus. Many viral infectious diseases including the current COVID-19 have an incident of exposure to the virus and a certain incubation period before the suspected patient begins to show some signs of infection (if any). The SEIR and SEIRD model are designed to account for this exposure rate by explicitly introducing a new variable ‘e(t)’ and corresponding parameter (β) in between the susceptible and infected population group to the SIR(D) model. Therefore, the SEIR model is of the form:

(3) $$\begin{array}{c} s\prime \left[t\right]=-\alpha i\left[t\right]s\left[t\right]\\ e\prime \left[t\right]=-\beta e\left[t\right]+\text{ αi}\left[t\right]s\left[t\right]\\ i\prime \left[t\right]=\text{ βe}\left[t\right]-\text{ γi}\left[t\right]\\ r\prime \left[t\right]=\text{ γi}\left[t\right] \end{array}$$

while the SEIRD model can be written as:

(4) $$\begin{array}{c} s\prime \left[t\right]=-\alpha i\left[t\right]s\left[t\right]\\ e\prime \left[t\right]=-\beta e\left[t\right]+\text{ αi}\left[t\right]s\left[t\right]\\ i\prime \left[t\right]=\text{ βe}\left[t\right]-\gamma i\left[t\right]-\text{ δi}\left[t\right]\\ r\prime \left[t\right]=\text{ γi}\left[t\right]\\ d\prime \left[t\right]=\text{ δi}\left[t\right] \end{array}$$

where d(t) is the number of those who die because of the virus infection and δ > 0 is the death rate associated with the COVID-19 [10].

**Figure 2.**
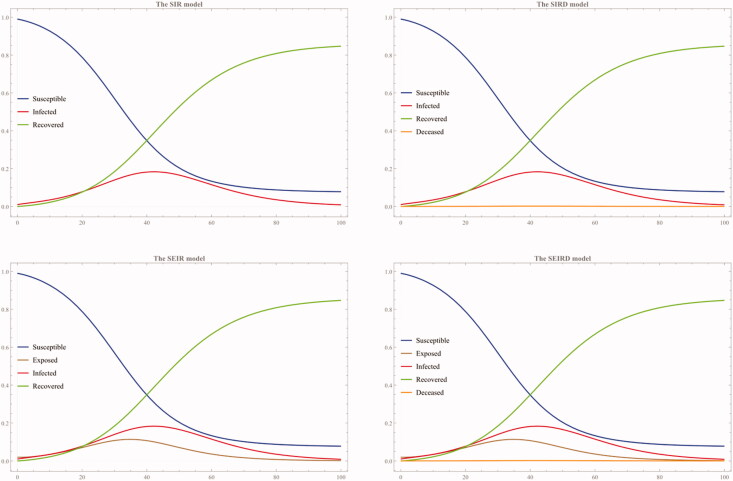
The first simulation with α = 0.2, β = 0.1, γ = 0.01, and δ = 0.01.

**Figure 3.**
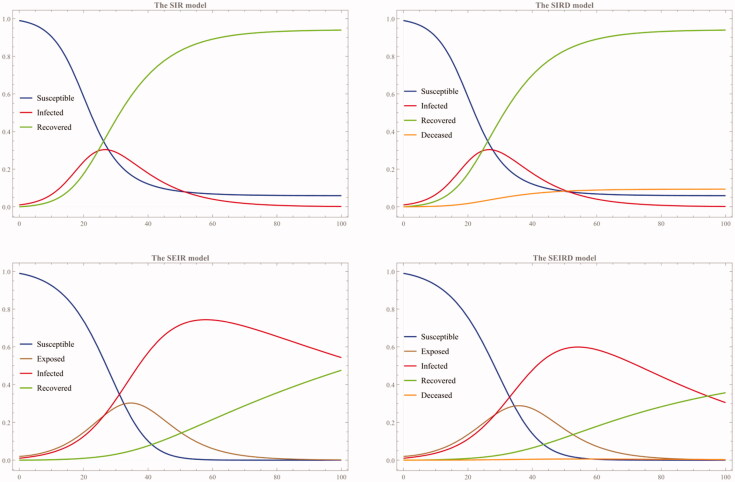
The second simulation with α = 0.3, β = 0.1, γ = 0.01, and δ = 0.01.

We can simulate these four models by assigning an arbitrary value to each parameter. For example, let us use the following arbitrary values assigned to each parameter and examine how solution curves of the respective system behave: (1) α = 0.2, β = 0.1, γ = 0.01, and δ = 0.01, and (2) α = 0.3, β = 0.1, γ = 0.01, and δ = 0.01 (See Figures 2 and 3).

As these two simulations show, the susceptible population decreases as more and more people are exposed to the virus and become infected. Some portions of the infected population recover, while the other sub-group die. Before this infection occurs, there is a certain exposure rate and/or incubation period that precedes the infection is confirmed, as shown in the second panel of both the SEIR and SEIRD model.

The second simulation (Figure 3) shows that the susceptible population decreases faster than the first case (Figure 2) as the infection rate α is set to be higher (0.3) than the first case (0.2). This higher infection rate is also reflected in the higher exposure rate curve. In the second panel of Figure 3 that shows both the SEIR and SEIRD model simulation result, the recovery rate curve is flattened at the beginning and only steadily rises towards the end of the simulation because of both higher exposure and infection rates.

### 2.3. Methods and data

The paper primarily relies on both the SEIR and SEIRD model to conduct a multi-stage parameter estimation, while occasionally compares the result from the SIR(D) model. As will become clear, the estimation result from both the SEIR and SEIRD model is far superior to what we can get from the SIR(D) model in particular epidemic contexts. One novel feature of this paper is to conduct a multi-stage parameter estimation using these models to identify potentially time-varying and context dependent parameters in each stage of the COVID-19 epidemic in Korea.

For this statistical analysis, the paper uses a manually compiled dataset taken from the official website of Korea Disease Control and Prevention Agency (KDCA). The KDCA has released various data related to the COVID-19 epidemic since the first infection case is confirmed. Individual researchers can view the daily press release and manually compile time series for the confirmed cases, recovered cases, deceased cases, all classified by sex, selected age group, and detailed geographical location of infection [11].

## 3. The result of multi-stage estimations and interpretation

### 3.1. The whole period

This section reports the multi-stage estimation results and offers an interpretation of some computed statistics such as the average days for recovery and the reproduction ratio in each stage.

Let us begin with the estimated SIR(D) parameters for the whole period from 18 February 2020 to 08 February 2021. The following table shows the SIR(D) model-based estimation result (See Table 1):

**Table 1.** The SIR(D) model parameter for the whole period (18 February 2020–08 February 2021).

	The SIR model				The SIRD model			
	Estimate	Standard Error	t-Statistic	p-Value	Estimate	Standard Error	t-Statistic	p Value
Susceptible population	126695	9087.6	13.9416	3.1343 × 10^ − 39^	126697	7419.9	17.0752	5.5971 × 10^ − 58^
Infection rate	0.1022	0.0056	18.3695	5.7165 × 10^ − 62^	0.1022	0.0045	22.4961	4.9314 × 10^ − 92^
Recovery rate	0.0658	0.0060	10.9122	9.7066 × 10^ − 26^	0.0658	0.0049	13.3640	8.8895 × 10^ − 38^
Fatality rate	–	–	–	–	0.0014	0.0008	1.5727	0.1161
R-Squared	0.851457				0.851453			
Reproduction ratio (Rt)	1.56				1.52			
Avg. days for recovery	15.20				15.20			

**Table 2.** The SIR(D) and SEIR(D) model parameter for the first wave (18 February – 15 June 2020).

	The SIR model				The SIRD model			
	Estimate	Standard Error	t-Statistic	p Value	Estimate	Standard Error	t-Statistic	p Value
Susceptible population	10521.4	83.0694	126.658	4.3367 × 10^−221^	10521.2	67.8089	155.1588	0
Infection rate	0.5836	0.0074	78.8855	3.9879 × 10^−173^	0.5836	0.0060	96.6826	3.9234 × 10^−259^
Recovery rate	0.0321	0.0006	53.5264	4.5938 × 10^−135^	0.0321	0.0060	13.3646	6.3533 × 10^−202^
Fatality rate	–	–	–	–	0.0009	0.0000	4.0723	0.00005
R-Squared	0.986975				0.986972			
AIC	3842.6				5615.0			
Reproduction ratio (Rt)	18.2				17.75			
Avg. days for recovery	31.2				31.2			
	The SEIR model				The SEIRD model			
	Estimate	Standard Error	t-Statistic	P-Value	Estimate	Standard Error	t-Statistic	P-Value
Susceptible population	10778	79.5510	135.4919	2.9343 × 10^−227^	9948	448.8807	22.1613	3.9988 × 10^−69^
Exposure/incubation rate	4.9500	0.4765	10.3890	4.3136 × 10^−21^	3.9457	0.5925	6.6597	1.0347 × 10^−10^
Infection rate	0.1294	0.0089	14.5105	1.3246 × 10^−34^	0.1596	0.0225	7.0842	7.4667 × 10^−12^
Recovery rate	0.0326	0.0006	58.9766	4.9859 × 10^−144^	0.0328	0.0006	58.5169	2.3680 × 10^−185^
Fatality rate	–	–	–		−0.0032	0.0017	−1.9036	0.0578
R-Squared	0.990524				0.990063			
AIC	3767.62				5518.68			
Reproduction ratio (Rt)	3.98				5.38			
Avg. days for recovery	30.71				30.45			

This parameter table shows that the estimated parameters are very sensitive to the number of variables and both effective reproduction ratio and average days for recovery derived from the parameters also vary depending on the number of variables. The estimated parameters look generally okay from a pure statistical point of view as relatively low and reasonable P-Values indicate. The computed average reproduction ratio is about 1.5 and the duration for the recovery is about 15 days, which are consistent with many international comparative studies, including the KDCA’s own computation.

**Figure 4.**
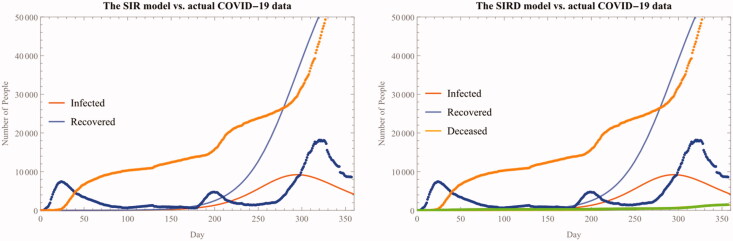
The SIR(D) fitted model for the whole period.

However, this estimation result is not robust in the sense that it is biased towards the latest development in the COVID-19 epidemic. Because of higher numbers of both confirmed and recovered cases concentrated in the latest third wave, the estimated infection and recovery rate parameters substantially underestimate the actual cases occurred during the first two waves. This estimation error in both SIR and SIRD model is also reflected in the relatively low R-Squared statistics (0.8515) (See Figure 4).

**Figure 5.**
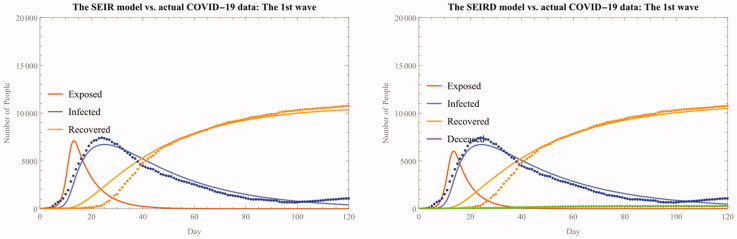
The SEIR(D) fitted model for the first wave.

In a sense, this biased estimation result is inevitable because the parameters from fitted models are taking the average of the cases without considering multiple waves present in the observed data. To put it another way, the very existence of multiple waves undermines the predictive power and usefulness of the SIR(D) model that is fundamentally based upon the assumption of ‘invariant’ and ‘uniform’ parameters. For this reason, we should carefully account for the presence of multiple waves when estimating parameters. The sub-section below shows how this careful usage of models and multi-stage analysis can be done.

### 3.2. The first wave

As we introduced earlier, the first wave began 30 days after the first imported infection case was detected and was ultimately contained 150 days later (from 18 February to 15 June 2020). On Feb. 29, new daily confirmed cases reached its peak of 909 and gradually fall thereafter.

The statistical analysis of the data for this period shows that both the infection rate and the basic reproduction ratio are much higher than the average of the whole period with more accurate model fit (See Table 2 and Figure 5). However, there are two statistical idiosyncrasies, one of which being the exceptionally high reproduction ratio, reaching 18.2 (SIR) and 17.8 (SIRD).

According to one meta-analysis of early studies conducted using initial period of Chinese COVID-19 data indicates that the mean value of the reproduction ratio is 3.38 ± 1.40 with a highest ratio being 6.49 [12]. Compared to this mean value of the reproduction ratio, both 18.2 and 17.8 are far higher. In addition, the average recovery day during this period is also very long (31.2 days in both SIR and SIRD model).

Though puzzling at first glance, we can easily resolve these problems by considering how the case definition was made during this period. The KDCA took an extremely cautious approach when it began to reclassify infected patients into the recovered group during the early stage of the epidemic. Since Korean health officials did not know much about the epidemiological and clinical nature of the COVID-19 at the beginning, they needed more time than usual to reclassify and discharge infected patients.

This delayed case definition naturally affects the number of active infection cases, lengthening the average duration for the recovery. The same delayed case classification brings down the recovery rate, while overstating the infection rate. Exceptionally high reproduction ratios specifically captured by both the SIR and SIRD model, therefore, are a direct result of this policy-induced low recovery rate, which is, in turn, determined by the KDCA’s extremely cautious reclassification criteria used during the early stage of the epidemic (For KDCA’s COVID-19 case definition, see [13]).

### 3.3. The second wave

The second wave was also triggered by a super spreader event on 15 August 2020, when Christian fundamentalists and conservative opposition party held a massive political demonstration in downtown Seoul. During this wave, some rally participant-cum-suspected patients fiercely resisted cooperating with public health officials, making it extremely difficult for the authority to properly conduct its contact tracing and other mitigation measures. Citing their non-cooperative behaviours, one may even argue that the second wave has never been fully suppressed, ultimately paving the way for the third wave that immediately followed [14].

Having these considerations in mind, let us examine estimated parameters in Table 3. The parameters for the SIR(D) show the similar pattern observed in the first wave: The reproduction ratio is consistently high (the SIR 6.4 and the SIRD 6.3), compared to the same ratio taken from both the SEIR (1.3) and the SEIRD model (4.7). This relatively high reproduction ratio captured by the SIR(D) may show the intensity of the infection during this period.

**Table 3.** The SIR(D) and SEIR(D) model parameter for the second wave (06 August– 04 October 2020).

	The SIR model				The SIRD model			
	Estimate	Standard Error	t-Statistic	p Value	Estimate	Standard Error	t-Statistic	p Value
Susceptible population	8417.74	117.6794	71.5312	2.0429 × 10^−98^	8417.78	95.9564	87.7251	3.4240 × 10^−147^
Infection rate	0.4580	0.0044	104.2026	3.1187 × 10^−117^	0.4581	0.00358	127.7970	1.6932 × 10^−175^
Recovery rate	0.0715	0.0023	32.5680	1.6718 × 10^−60^	0.0715	0.00179	39.9410	3.6041 × 10^−90^
Fatality rate	–	–	–	–	0.0009	0.00179	1.0904	0.2770
R-Squared	0.981698				0.981698			
AIC	1836.82				3515.46			
Reproduction ratio (Rt)	6.41				6.33			
Avg. days for recovery	13.98				13.98			
	The SEIR model				The SEIRD model			
	Estimate	Standard Error	t-Statistic	P-Value	Estimate	Standard Error	t-Statistic	P-Value
Susceptible population	9283.76	195.8409	47.4046	9.0170 × 10^−78^	6280.18	256.7824	24.4572	2.3841 × 10^−58^
Exposure/incubation rate	3.4854	0.3660	9.5222	3.1274 × 10^−16^	1.5170	0.1038	14.6084	4.0849 × 10^−32^
Infection rate	0.0931	0.0084	11.0398	8.3316 × 10^−20^	0.2203	0.0184	11.9841	1.5113 × 10^−24^
Recovery rate	0.0717	0.0018	39.5476	3.5845 × 10^−69^	0.0728	0.0016	46.5140	1.9031 × 10^−100^
Fatality rate	–	–	–	–	−0.0264	0.0030	−8.7688	1.5549 × 10^−15^
R-Squared	0.988878				0.987295			
AIC	1779.09				2616.59			
Reproduction ratio (Rt)	1.30				4.76			
Avg. days for recovery	13.95				13.75			

Compared to the first wave, however, we have about ‘14 days of average recovery period,’ regardless of the type of the model used in this period. This shorter average recovery day in the second wave (and the third wave as well below) is more to do with the revised case definition that the Korean health authority begins to use towards the end of the first wave, rather than any changes in pathogenic properties of the novel coronavirus.

**Figure 6.**
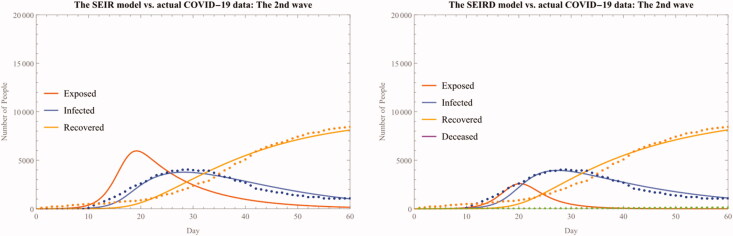
The SEIR(D) fitted model for the second wave.

We can also confirm that the accuracy of the model parameters captured by both R-Squared and AIC across the model has been substantially improved from the whole period analysis (See also Figure 6).

### 3.4. The third wave

Starting from around mid-October, daily confirmed cases began to rise again, marking the beginning of the third and concurrent wave in the COVID-19 epidemic. Table 4 tabulates estimated parameters showing the severity of the concurrent wave of the COVID-19 epidemic (See Table 4).

**Table 4.** The SIR(D) and SEIR(D) model parameters for the third wave (05 October 2020 – 08 February 2021).

	The SIR model				The SIRD model			
	Estimate	Standard Error	t-Statistic	p Value	Estimate	Standard Error	t-Statistic	p Value
Susceptible population	54762.8	681.1289	80.3999	3.7442 × 10^−181^	54762.0	555.7720	98.5334	3.6515 × 10^−271^
Infection rate	0.1989	0.0012	164.3275	1.6413 × 10^−257^	0.1988	0.0009	201.3749	0
Recovery rate	0.0632	0.0016	38.80145	4.8860 × 10^−108^	0.0632	0.0013	47.5489	2.3982 × 10^−161^
Fatality rate	–	–	–	–	0.0014	0.0004	3.0233	0.0027
R-Squared	0.982381				0.982386			
AIC	4643.84				6809.35			
Reproduction ratio (Rt)	3.15				3.08			
Avg. days for recovery	15.82				15.82			
	The SEIR model				The SEIRD model			
	Estimate	Standard Error	t-Statistic	P-Value	Estimate	Standard Error	t-Statistic	P-Value
Susceptible population	53087.5	1234.4408	43.0053	1.6184 × 10^−117^	37263.0	92.6018	402.4013	0
Exposure/incubation rate	0.6831	0.0685	9.9691	6.3250 × 10^−20^	0.8173	0.00267	305.7060	1.1364 × 10^−322^
Infection rate	0.0664	0.0086	7.7648	2.1016 × 10^−13^	0.0490	0.0008	63.0770	3.9189 × 10^−155^
Recovery rate	0.0622	0.0016	39.8060	3.4382 × 10^−110^	0.0621	0.0014	43.8430	4.3340 × 10^−119^
Fatality rate	–	–	–	–	−0.0255	0.0009	−25.9032	1.2816 × 10^−72^
R-Squared	0.98334				0.987227			
AIC	4631.64				4566.18			
Reproduction ratio (Rt)	1.07				1.34			
Avg. days for recovery	16.07				16.10			

One interesting characteristics of the latest wave is that the absolute number of confirmed, recovered, and deceased cases are far higher than the prior two waves. Nonetheless, the estimated parameters and computed statistics (especially, the reproduction ratio) are not comparably higher. The simple reason is that the confirmed, recovered, and deceased cases in the third wave started off with higher initial values than the first two waves. Therefore, even when the peak number of daily confirmed cases once reached more than 1241 (on 25 December 2020), the single highest daily confirmed case number in the entire period of the COVID-19 epidemic, the average reproduction ratios are not comparably higher than those in the previous two waves.

**Figure 7.**
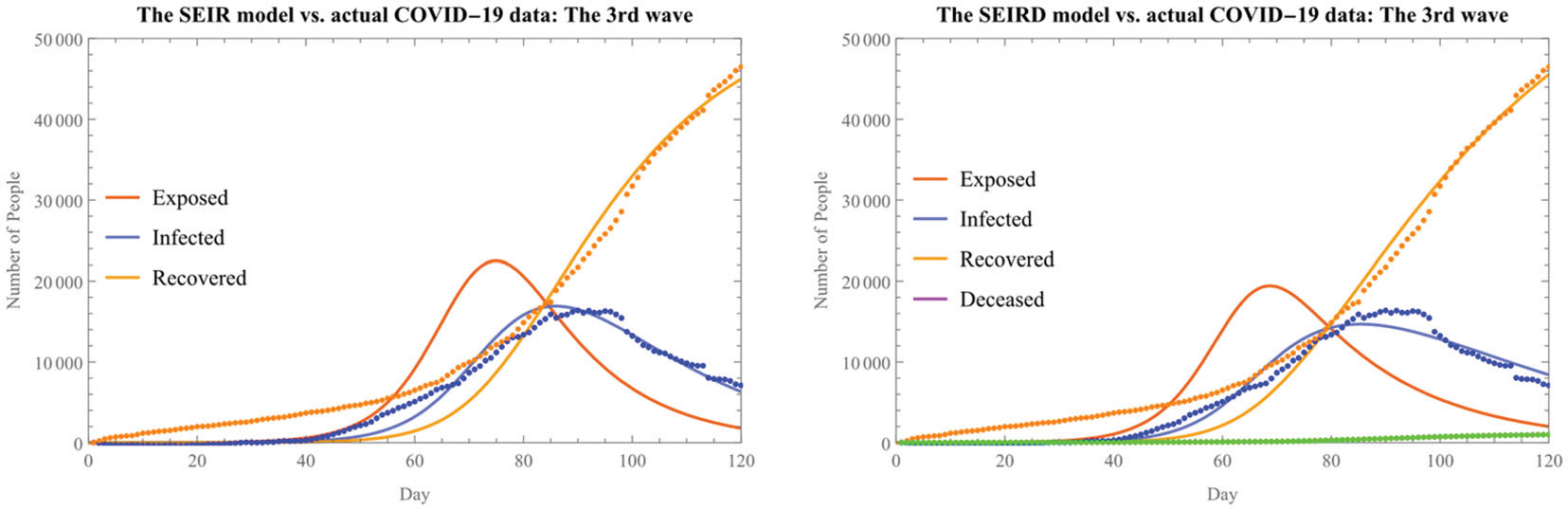
The SEIR(D) fitted model for the concurrent third wave.

It is also notable to see that there is no single definitive criterion that we should use to select a particular model other than the purely statistical model selection criterion. Both the SIR(D) models are equally sound as their counterparts, the SEIR(D) models, in terms of their estimated parameters and derived statistics. The computed P-Values and reproduction ratios are equally reasonable to use regardless of the model, and the AIC also shows that they are close to each other irrespective of the models used (See also Figure 7).

## 4. Discussion and limitation

The COVID-19 epidemic in Korea has exhibited multiple stages of its development, whose immediate causes and transmission patterns differ from one another. This paper has conducted multi-period statistical analysis based upon both the SIR(D) and SEIR(D) models to capture this time-varying and context-dependent transmission dynamics more accurately. It is demonstrated that the SIR-based epidemic models are still useful and informative, but only when they are used to carefully account for the presence of multiple waves of the COVID-19 epidemic.

This multi-stage estimation of the model parameters has shown that both transmission rate and the basic reproduction ratio can rise substantially in the absence of the government’s effective non-pharmaceutical interventions. At the same time, even if the public health authority is willing to implement timely and proper public health measures, the success of these policies is largely dependent on how the public respond to the proposed measures. The value of estimated parameters and computed statistics that we have examined above are the reflection of the aggregate outcome of these interactions, and consistently higher infection rate parameters and reproduction ratios across the model appeared during the second wave seem to show the limited effectiveness of non-pharmaceutical interventions in the face of fierce opposition.

With respect to statistical analysis, it is challenging to identify the single best epidemic model sorely relying on any single model selection criterion. The SIR-based epidemic models are a good starting point. But the SIR(D) model fails to generate robust parameters especially when they are used to cover the entire period of the epidemic. For this reason, this paper has attempted to identify multiple waves of the epidemic and estimate model parameters for each wave to find time-varying and context-dependent transmission dynamics in each stage.

Even on this ground, however, the epidemic model and the statistical analysis cannot fully address the data problem associated with the official case definition and the measurement error we faced during the first wave of the epidemic. The delayed recovery case definition brought down the average recovery rate parameter, thereby inducing higher estimated infection rate and lengthening average days for recovery during the first wave.

Nonetheless, a careful application of epidemic models and multi-stage statistical analysis based upon the proposed models is far superior to a blind usage of the same epidemic models for the whole period analysis because the former shows time-varying and context-dependent transmission dynamics of the COVID-19 epidemic more accurately.

## 5. Conclusion

The paper discusses multi-period estimation of the COVID-19 epidemic data for Korea based upon the selected SIR epidemic models, while emphasising the importance of finding time-varying transmission dynamics of the novel coronavirus. For this purpose, the paper attempts to identify major stages of the COVID-19 epidemic in Korea and uses a selected SIR-based epidemic models to estimate the parameters that may capture evolutionary aspects of the COVID-19 epidemic in this country.

From a theoretical point of view, the analysis in this paper points to the limited usefulness of the SIR-based epidemic models. Particularly, the ‘invariant parameter’ assumption shared by these epidemic models is questioned. The SIR models and their parameters can be grossly misleading if they are not accompanied by proper considerations of the given context, in which the model is being used. As an alternative, the paper attempts to show that we can better utilise the same SIR epidemic models by carefully accounting for each distinctive stage of the epidemic.

This multi-stage statistical analysis reveals that the transmission dynamics of the novel coronavirus changes, primarily depending on how effectively the government’s non-pharmaceutical interventions work. The multi-stage estimation of model parameters and derived statistics can capture the time-varying relative effectiveness of and challenges to the government’s mitigation strategies in each stage.

## Data Availability

The dataset compiled and used in this paper is made publicly available from the author’s personal repository at https://github.com/interglobe07/COVID-19-Data-Korea and is being regularly updated.
